# Screening of Barley Resistance Against Powdery Mildew by Simultaneous High-Throughput Enzyme Activity Signature Profiling and Multispectral Imaging

**DOI:** 10.3389/fpls.2018.01074

**Published:** 2018-07-23

**Authors:** Matheus T. Kuska, Jan Behmann, Dominik K. Großkinsky, Thomas Roitsch, Anne-Katrin Mahlein

**Affiliations:** ^1^Institute for Crop Science and Resource Conservation-Plant Diseases and Plant Protection, University of Bonn, Bonn, Germany; ^2^Department of Plant and Environmental Sciences, Copenhagen Plant Science Centre, University of Copenhagen, Frederiksberg, Denmark; ^3^Department of Plant and Environmental Sciences, Copenhagen Plant Science Centre, University of Copenhagen, Taastrup, Denmark; ^4^Institute of Sugar Beet Research (IfZ), Göttingen, Germany

**Keywords:** crop resistance, phenotyping, multispectral imaging, invertase, *Blumeria graminis* f.sp. *hordei*, PhenoLab, classification, support vector machine

## Abstract

Molecular marker analysis allow for a rapid and advanced pre-selection and resistance screenings in plant breeding processes. During the phenotyping process, optical sensors have proved their potential to determine and assess the function of the genotype of the breeding material. Thereby, biomarkers for specific disease resistance traits provide valuable information for calibrating optical sensor approaches during early plant-pathogen interactions. In this context, the combination of physiological, metabolic phenotyping and phenomic profiles could establish efficient identification and quantification of relevant genotypes within breeding processes. Experiments were conducted with near-isogenic lines of *H. vulgare* (susceptible, mildew locus o *(mlo)* and Mildew locus a *(Mla)* resistant). Multispectral imaging of barley plants was daily conducted 0–8 days after inoculation (dai) in a high-throughput facility with 10 wavelength bands from 400 to 1,000 nm. In parallel, the temporal dynamics of the activities of invertase isoenzymes, as key sink specific enzymes that irreversibly cleave the transport sugar sucrose into the hexose monomers, were profiled in a semi high-throughput approach. The activities of cell wall, cytosolic and vacuole invertase revealed specific dynamics of the activity signatures for susceptible genotypes and genotypes with *mlo* and *Mla* based resistances 0–120 hours after inoculation (hai). These patterns could be used to differentiate between interaction types and revealed an early influence of *Blumeria graminis* f.sp. *hordei (Bgh)* conidia on the specific invertase activity already 0.5 hai. During this early powdery mildew pathogenesis, the reflectance intensity increased in the blue bands and at 690 nm. The *Mla* resistant plants showed an increased reflectance at 680 and 710 nm and a decreased reflectance in the near infrared bands from 3 dai. Applying a Support Vector Machine classification as a supervised machine learning approach, the pixelwise identification and quantification of powdery mildew diseased barley tissue and hypersensitive response spots were established. This enables an automatic identification of the barley-powdery mildew interaction. The study established a proof-of-concept for plant resistance phenotyping with multispectral imaging in high-throughput. The combination of invertase analysis and multispectral imaging showed to be a complementing validation system. This will provide a deeper understanding of optical data and its implementation into disease resistance screening.

## 1. Introduction

The ascomycota *Blumeria graminis* f.sp. *hordei* (*Bgh*) is an obligate biotroph organism and the causal agent of barley's powdery mildew. It infests leaves and all green parts of barley plants. After the pre-penetration stage which finished with the penetration of the epidermal cell 15 hai, the post-penetration stage starts with an invagination of the fungus inside the epidermal cell. After this entering, *Bgh* develops a haustorium in the periplasmatic space 24 hai (Francis et al., 1996; Pryce et al., 1999). Haustoria are the feeding organs of *Bgh* and they deliver nutrients and necessary compounds for the biotrophic fungus (Green et al., 2002). A missing ATPase activity in *Bgh* is predicted to cause a loss of solute retention capacity of the host cell, which enable *Bgh* to take up nutrients (Gay et al., 1987). To make necessary carbohydrates available, *Bgh* reduce the activity of Ribulose-1,5-bisphosphate carboxylase/oxygenase (RuBisCO) and further enzymes of the Calvin cycle (Scholes et al., 1994; Wright et al., 1995a). Studies with powdery mildew of wheat (*B. graminis* f.sp. *tritici*) indicated that powdery mildew triggers the accumulation of acid invertases to change the source-sink relation in cereals (Wright et al., 1995b). With the nutrient income, *Bgh* is able to develop secondary mycelium on the leaf surface. Close to the area of the primary haustorium, conidiophores are grown, producing new conidia 5 dai. The disease is then macroscopically visible as white pustules and the conidiophores produce ~6,000 conidia per millimeter per day (Blumer, 1967). Thus fungal plant pathogens have an strong impact on gen-protein-hormone-metabolite signaling and on the cell histology of plants to overcome resistances such as waxy cuticula, cell wall and the innate resistance. Resistant barley genotypes are typically incompatible plant-pathogen systems e.g., based on mildew locus o (*mlo*, papilla formation) or mildew locus a (*Mla*, hypersensitive response resistance) (Jørgensen, 1992). Different plant-pathogen interactions have specific impact on the plant physiology and histology which individually influence the spectral reflectance signature of plants (Mahlein et al., 2012; Wahabzada et al., 2015).

To determine and assess these changes, different optical sensors were established which non-invasively measure specific spectral ranges e.g., spectral sensors, chlorophyll fluorescence and thermography (Mahlein, 2016). These optical sensors record the plant phenotype. Phenotyping is the visual description and assessment from single organs to the canopy, and this phenotype is influenced by the genome and the environment (Fiorani and Schurr, 2013). In this context, plant spectral reflectance from 380 to 2,500 nm can be measured using hyperspectral imaging. The recorded reflectance signature can be used to assess the plant health status, because several chemical compounds and the cell structure has specific optical characteristics. The visual range (VIS, 400–700 nm) is mainly influenced by photo pigments like chlorophyll, carotenoide and anthocyanin (Gitelson et al., 2001; Blackburn, 2007). The near infrared (NIR, 700–1,000 nm) is characterized by scattering processes of the plant and on the leaf structure. Spectral range from 1,000 to 2,500 nm is described as the short wave infrared range (SWIR) with specific water absorption bands (Whiting et al., 2004). During plant pathogenesis the characteristic spectral signature pattern is specifically changed over time (Mahlein et al., 2010). These changes in the spectral reflectance intensity and spectral pattern can be also used to derive histological changes and biological stages of the plant and pathogenesis (Wahabzada et al., 2016). This enables the characterization of the causal agent and the pathogenesis stage (Mahlein et al., 2012). According to these findings, different plant-pathogen interactions specifically influence the spectral signature and the detection of resistant and tolerant crop varieties may be possible. Recent studies identified different resistance reactions such as barley resistances against powdery mildew or sugar beet lines resistant against *Cercospora* leaf spot using hyperspectral imaging (Kuska et al., 2017; Leucker et al., 2017). Spectral pattern of the corresponding pathogenesis differ and can be distinguished from incompatible plant-pathogen interactions (Arens et al., 2016; Oerke et al., 2016; Kuska et al., 2017; Leucker et al., 2017). Therefore, hyper-/multispectral imaging is a promising technique for high-throughput phenotyping approaches in plant resistance breeding with increasing flexibility, due to technical and methodology developments (Behmann et al., 2018; Thomas et al., 2018b). Leaf chemicals and metabolites can be detected using hyperspectral imaging and machine learning approaches (Arens et al., 2016; Pandey et al., 2017), but the relationship of biochemical mechanisms and hyperspectral reflectance during plant-pathogen interactions are not completely clear.

Currently, many crop improvements are based on molecular plant breeding techniques to identify key factors (Wenzel, 2006; Schaart et al., 2016). Established molecular markers and genetic maps are used for marker-assisted selection, efficient parental selection and high-throughput screening of desired genotypes (Wenzel, 2006). In this context, plant invertases play a key role in plant development, cell regulation, metabolism, hormone signaling and defense response (Roitsch and González, 2004; Proels and Hückelhoven, 2014). The proposed main function of invertases is the carbohydrate partitioning, but investigations of the last decades revealed the multi-functionality of invertases (Roitsch and González, 2004; Proels and Hückelhoven, 2014). Invertases irreversibly cleaves sucrose to glucose and fructose which are the major transported sugars in higher plants (Williams et al., 2000). These can be taken up by plant cells due to hexose transporters (Roitsch, 1999). A coordinated regulation of primary metabolism and pathogen defense responses has been shown (Ehness and Roitsch, 1997; Berger et al., 2007). Studies by Roitsch et al. (2003), Proels and Hückelhoven (2014), and Tauzin and Giardina (2014) reviewed functions of cell wall invertases (Cw-Inv) in the context of pathogen infection. They highlighted the modes of Cw-Inv in plant cell regulations and plant-pathogen interactions. In addition to the extracellular invertase isoenzyme, two intracellular isoenzymes were shown to be involved in the infection by necrotrophic fungi (Berger et al., 2004) and hemibiotrophic bacteria (Bonfig et al., 2010). Such sugar-based signals were also shown in barley-powdery mildew compatible and incompatible interactions (Scholes et al., 1994; Swarbrick et al., 2006). Scholes et al. (1994) hypothesized that apoplastic invertase increased in barley during powdery mildew pathogenesis, because of a increased activity of acid invertase. These specific enzyme kinetics could be used as possible biomarkers for the detection of resistant plant genotypes. Linking these physiological and the optical scales will improve the performance of hyper-/multispectral imaging in plant resistance breeding and will establish a new non-invasive methodology for plant sciences (Großkinsky et al., 2015, 2017).

In this study, different barley genotypes were measured in a high-throughput approach using multispectral imaging. In the first experiment, plants were not inoculated to determine the natural senescence of the genotypes and the influence on the spectral reflectance signature. In the second experiment, plants were inoculated with *Bgh*. Powdery mildew pathogenesis as well as *mlo* gene-based resistances and *Mla* gene-based resistances were identified. Data analysis approach from machine learning could establish and validate resistance phenotyping by multispectral imaging. This is the basis for an automated spectral characterization of susceptible and resistance phenotypes in high-throughput. Furthermore, the temporal dynamics of changes in the activities of invertase isoenzymes were analyzed during different barley-powdery mildew interactions. Proved barley-powdery mildew interactions could be identified by invertase activity pattern already from 0.5 hai.

## 2. Materials and methods

### 2.1. Plant cultivation and inoculation of powdery mildew

The experimental set-up was divided into two parts. For both investigations, plants were grown in commercial substrate (SW Horto AB, Hammenhög, Denmark) for 10 days in the greenhouse at 22/18°C and a photoperiod of 16 h. *H. vulgare* cv. Ingrid wild type (WT) was used as a susceptible genotype to powdery mildew. The corresponding near-isogenic line Ingrid M.C. 20, containing dysfunction in mildew locus o 3 (*mlo3*) (Hinze et al., 1991) and near-isogenic line cv. Pallas 22, containing dysfunction in *mlo5* gene were used to assess non race-specific papilla based resistance. *H. vulgare* cv. Ingrid I10 with resistant mildew locus a 12 (*Mla12*) and Pallas 01 with *Mla1* and *Mla12* resistance loci were used to analyze a hypersensitive response (Kølster et al., 1986; Boyd et al., 1995).

*Bgh*, isolate A6 is avirulent to cv. Ingrid M.C. 20 and I10, and cv. Pallas 01 and 22 (Wolter et al., 1993; An et al., 2006; Swarbrick et al., 2006) and was maintained on cv. Ingrid WT in a controlled environment. Twenty-four hours before inoculation the conidia of heavily infested plants were shaken off and discarded in order to assure homogenous and vital conidia for inoculation. For each genotype, 80 primary leaves were inoculated with a density of $\bar{X}$ = 307 (± 112) conidia/cm^2^ from young powdery mildew pustules (7–10 dai). Further 80 primary leaves were kept untreated (non-inoculated) as healthy control. For destructive measurements, five primary leaves of both treatments were sampled and frozen in liquid nitrogen 0.5, 12, 24, 48, 72, 96, and 120 hai.

### 2.2. Total protein extraction

Barley leaves were weighed and then homogenized in liquid nitrogen with 0.1 % PVPP. According to (Jammer et al., 2015), 1 ml extraction buffer (40 mM TRIS-HCl pH 7.6, 3 mM MgCl_2_, 1 mM EDTA, 0.1 mM PMSF, 1 mM benzamidine, 14 mM ß-mercaptoethanol, 24 μ M NADP) was mixed with 500 mg powdered material for 60 min at 4°C. The homogenate was centrifuged at 4°C and 20,000 g for 45 min. The supernatant was transferred into a new tube and kept on ice as a crude extract. According to Jammer et al. (2015), the remaining pellet was washed three times with ddH_2_O and resuspended in 1 ml high salt buffer (1 M NaCl, 40 mM TRIS-HCl pH = 7.6, 3 mM MgCl_2_ and 15 mM EDTA) over night at 4°C in a dark room. The resuspended pellet was centrifuged at 4°C and 20,000 g for 25 min. The supernatant was transferred into a new tube as the cell wall extract. To reduce the salt concentration, both extracts were dialysed overnight against 20 mM potassium phosphate buffer (pH = 7.4) at 4 °C in a dark room. Extracted protein content in both extracts was determined according to the Bradford method (Bradford, 1976), using BSA Fraction V as standard protein. The extracts were aliquoted, frozen in liquid nitrogen and stored at −20°C for further use.

### 2.3. Enzyme activity profiling

For semi-high-throughput analysis, a 96-well microtiter plate (Sarstedt, Nümbrecht, Germany) formate was used with a 5 μl citric acid-phosphate-buffer, 5 μl 0.1 M sucrose, 35 μl ddH_2_O, and 5 μl of dialysed protein extract. For determination of Cw-Inv activity, aliquots of the cell wall extract were incubated with citric acid-phosphate-buffer pH = 4.5 (454 mM Na_2_HPO_4_, 273 mM citric acid). For determination of cytosolic invertase (Cyt-Inv) activity, aliquots of the dialysed crude extract were incubated with citric acid-phosphate-buffer pH = 6.8 (772 mM Na_2_HPO_4_, 114 mM citric acid) and to test vacuolar invertase (Vac-Inv), citric acid-phosphate-buffer pH = 4.5 was used. In addition, a 0–50 nmol glucose standard curve was prepared with ddH_2_O. Reaction mixtures were incubated for 30 min at 37°C and then cooled down for 5 min on ice to stop the reaction. According to Jammer et al. (2015), the cooled down reaction mixtures were incubated with 200 μl of glucose oxidase-peroxidase reagent (10 U ml^−1^ GOD, 0.8 U ml^−1^ POD, 0.8 mg ml^−1^ ABTS, 0.1 M potassium phosphate buffer, pH = 7.0) for 30 min at 25°C. The amount of liberated glucose was determined by measuring the absorbance at 405 nm in a plate reader (Ascent Multiskan, Thermo Fisher Scientific, Waltham, USA). Specific activities were expressed as nkat gFW^−1^. All assays were carried out in triplicate and relative differences of nkat gFW^−1^ were calculated using Formula 1. To consider the biological dynamic, a variance propagation was calculated as measure of dispersion according to Formula 2.

**Formula 1**. Calculation of relative differences in specific activity *rD*[%] [nkat gFW^−1^] of invertases between non-inoculated (healthy) and *B. graminis* f.sp. *hordei* inoculated barley leaves.

$$\begin{array}{l} \frac{\left(inoculated-healthy\right)}{\left(healthy\right)}\times 100=rD \end{array}$$

**Formula 2**. Calculation of the standard deviation of relative activity differences between non-inoculated (healthy) and *B. graminis* f.sp. *hordei* inoculated barley leaves by variance propagation as measure of dispersion of relative difference specific activity rD[%] [nkat gFW^−1^] of invertases.

$$\begin{array}{l} \sqrt{\left({\left(\frac{1}{healthy}\right)}^{2}\times {\left(\frac{s_{inoculated}}{\sqrt{3}}\right)}^{2}+{\left(\frac{inoculated}{{\left(healthy\right)}^{2}}\right)}^{2}\times {\left(\frac{s_{healthy}}{\sqrt{3}}\right)}^{2}\right)}=s\left(rD\right) \end{array}$$

### 2.4. Multispectral image acquisition and data analysis

Using narrow banded LEDs, multispectral images with 10 wavelength bands were automatically acquired at spectral bands 365, 460, 525, 570, 645, 670, 700, 780, 890, and 970 nm and spatial resolution of five megapixels (PhenoLab, Videometer, Hørsholm, Denmark) (Svensgaard et al., 2014). A hemisphere setup (PhenoLab, Videometer, Hørsholm, Denmark) was used to assure homogeneous and diffuse illumination of the plants by high power LED sources. Multispectral images consist of consecutive panchromatic images each with a specific LED illumination at the corresponding wavelength. Plants were daily randomized and imaged 0–8 dai.

Spectral signatures of pixels from healthy and diseased regions were extracted manually. Therefore, a rectangular region of interest of ≥ 155 pixels was extracted. When a symptomatic area became visible the amount of pixels extracted increased depending on the symptom development. The spectral reflectance signature was calculated as the arithmetic average of the regions of interest.

For data driven analysis of the multispectral imaging data, a non-linear Support Vector Machine (SVM) classification with a radial base function kernel was applied. Two different classification models for powdery mildew symptoms and HR-spots were learned. As training data healthy plant pixels of the control group and manually selected powdery mildew symptoms and HR spots were used, respectively. To enhance accuracy at the last 2 days of the second experiment, healthy plant pixels provided by the *mlo3*-resistant genotype were included in the training data. Hyperparameters were optimized using the combination of ten-fold cross-validation and grid-search. Predictions were obtained applying the model on pixel-level to the plant pixels within the image. Background image parts like soil, tray and conveyor system were removed in a preprocessing step by thresholding and spatial masks. The pixel-wise classification was then summarized per tray to the ratio of affected pixels to all plant pixels expressed in percentage. As both models were applied to all images, two ratio values per day and image are derived.

### 2.5. Separability of phenotypes

Based on the ratio of powdery mildew symptoms, and HR-spots the plants were assigned to a response type, (I) susceptible, (II) *mlo* resistance or (III) *Mla* resistance. A threshold classification of the 20 samples was performed whereas each sample was represented by the two ratios determined by the SVM. A threshold of 5 % on the powdery mildew ratio was used to separate resistant and susceptible samples whereas a threshold of 0.45 % on the HR-ratio was used to separate *Mla* resistance and *mlo* resistance.

## 3. Results

### 3.1. Temporal dynamics of changes in activities of invertase isoenzymes during barley-powdery mildew interactions

**Figure 1:**
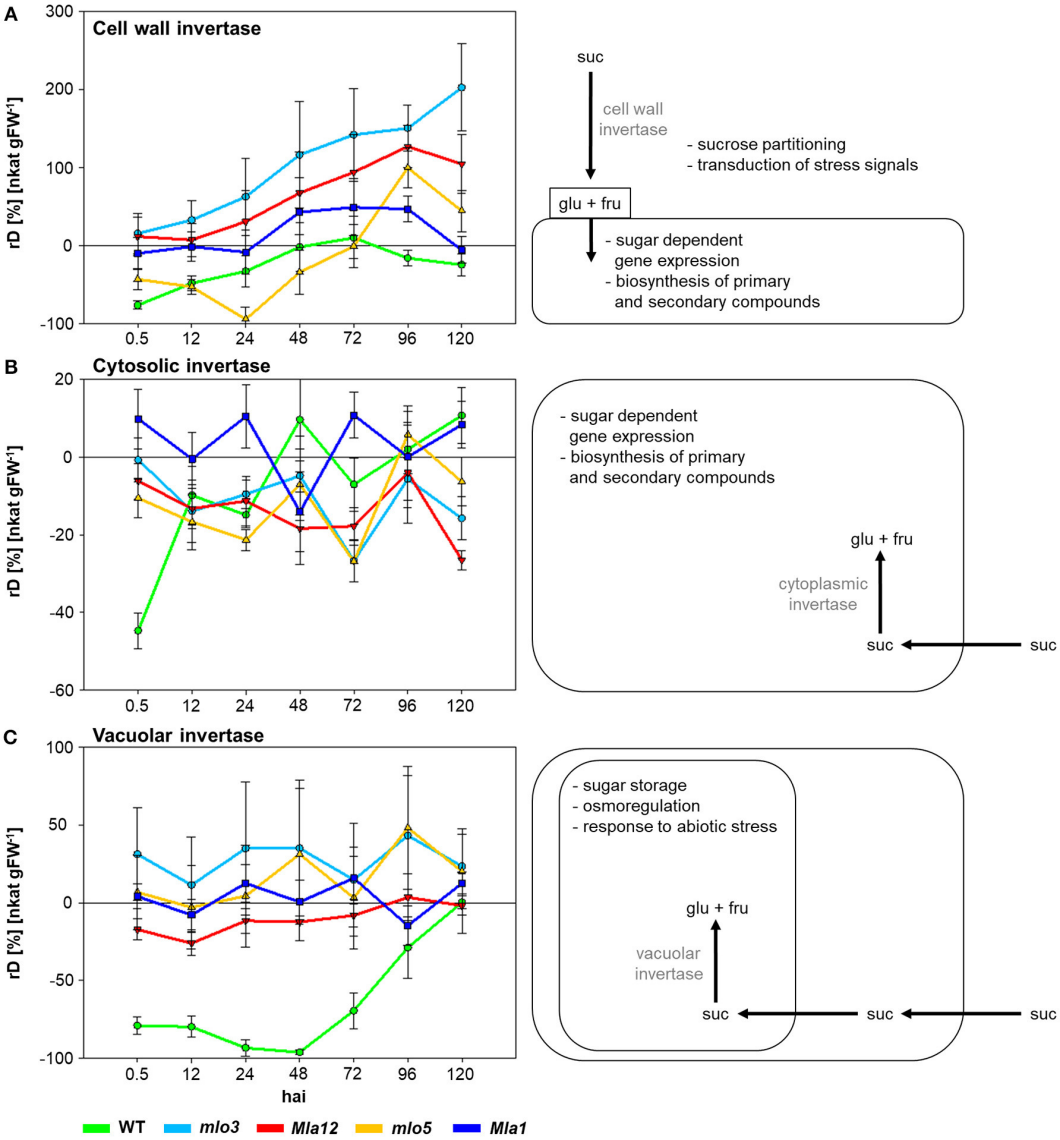
The effect of compatible and incompatible *mlo* and *Mla* barley interactions with *B. graminis* f.sp. *hordei* on the specific activity signatures of invertases 0.5–120 hai. Relative differences (rD) of the specific activity between the inoculated near-isogenic lines and their corresponding non-inoculated leaves were calculated. Positive values demonstrate higher invertase activity in inoculated leaves, negative values higher invertase activity of non-inoculated leaves. Each invertase shows a specific activity signature for each near-isogenic line. Data shown are from a representative of three independent experiments (*n* = 5 × 3 biological replicates × technical replicates).

To characterize susceptible and *Bgh* resistant genotypes the specific activities of cell wall, cytosolic and vacuolar invertases were analyzed (Figure 1). Therefore, relative differences (rD) of the specific activity of inoculated plants to non-inoculated plants were calculated. Positive values indicate higher specific activity in *Bgh* inoculated individuals compared to non-inoculated plants. Negative values showed a lower activity.

Cell wall invertase activity increased over the experimental period (Figure 1A). Susceptible WT showed the lowest activity of −77% comparing to the non-inoculated control 0.5 hai. The *mlo5* genotype showed a declined activity of the cell wall invertase until 24 hai. Highest activity up to 200% was measured in *mlo3* leaves. Cw-Inv activity revealed significant differences between *mlo3* and *mlo5* on all investigated time points in exception of 96 hai (Table 1). Both *Mla* genotypes showed a similar cell wall invertase activity pattern with significant differences 96 and 120 hai (Table 1).

**Table 1:** Hours after inoculation with *B. graminis* f.sp. *hordei* that have significant differences in invertase activity of proved near-isogenic barley lines (Welch's *t*-test, α = 0.05).

	**WT**	***mlo3***	***Mla12***	***mlo5***	***Mla1***
**Cw-Inv**					
WT	X	12, 24, 72, 96, 120	12, 96, 120	0, 24, 96, 120	0, 12, 96
*mlo3*	-	X	-	0, 12, 24, 48, 72, 120	96, 120
*Mla12*	-	-	X	0, 12, 24, 48, 72	96, 120
*mlo5*	-	-	-	X	12, 24, 96
*Mla1*	-	-	-	-	X
**Cyt-Inv**					
WT	X	0, 72, 120	0, 48, 120	0, 72, 120	0, 24, 72
*mlo3*	-	X	120	24	24, 72, 120
*Mla12*	-	-	X	-	0, 24, 72, 120
*mlo5*	-	-	-	X	0, 12, 24, 72, 120
*Mla1*	-	-	-	-	X
**Vac-Inv**					
WT	X	72, 96	0, 12, 24, 72	0, 12, 72, 96	0, 12, 24, 72
*mlo3*	-	X	-	-	-
*Mla12*	-	-	X	-	0, 24
*mlo5*	-	-	-	X	-
*Mla1*	-	-	-	-	X

Specific activity of WT cytosolic invertase increased after 0.5 hai (Figure 1B). Compared to control plants, the activity was higher at 48 and 120 hai. The *mlo3* and *mlo5* genotypes showed similar dynamics in the cytosolic invertase activity with an 6% increased activity in *mlo5* leaves 96 hai. But significant differences were shown to all other tested time-points (Table 1). *Mla1* had the highest cytosolic invertase activity up to 11% over the experimental period (Figure 1B). The *Mla12* genotype had a declined activity and could significantly differentiate from *Mla1* 0, 24, 72, and 120 hai (Table 1).

Susceptible WT had a declined activity of the vacuolar invertase to −96% until 48 hai (Figure 1C). Then, the activity normalized until the end of the analysis 120 hai. Interestingly, this activity was significantly different to all resistant barley near-isogenic lines (Table 1). Both *mlo* genotypes showed an similar dynamic pattern of the vacuolar invertase activity with the highest increase of 48% 96 hai (Figure 1C). HR based resistant *Mla12* genotype showed lower activity comparing to non-inoculated plants until 72 hai. *Mla1* plants had an increased activity at 24, 72, and 120 hai.

### 3.2. Influence of barley-powdery mildew interactions on the multispectral reflectance

**Figure 2:**
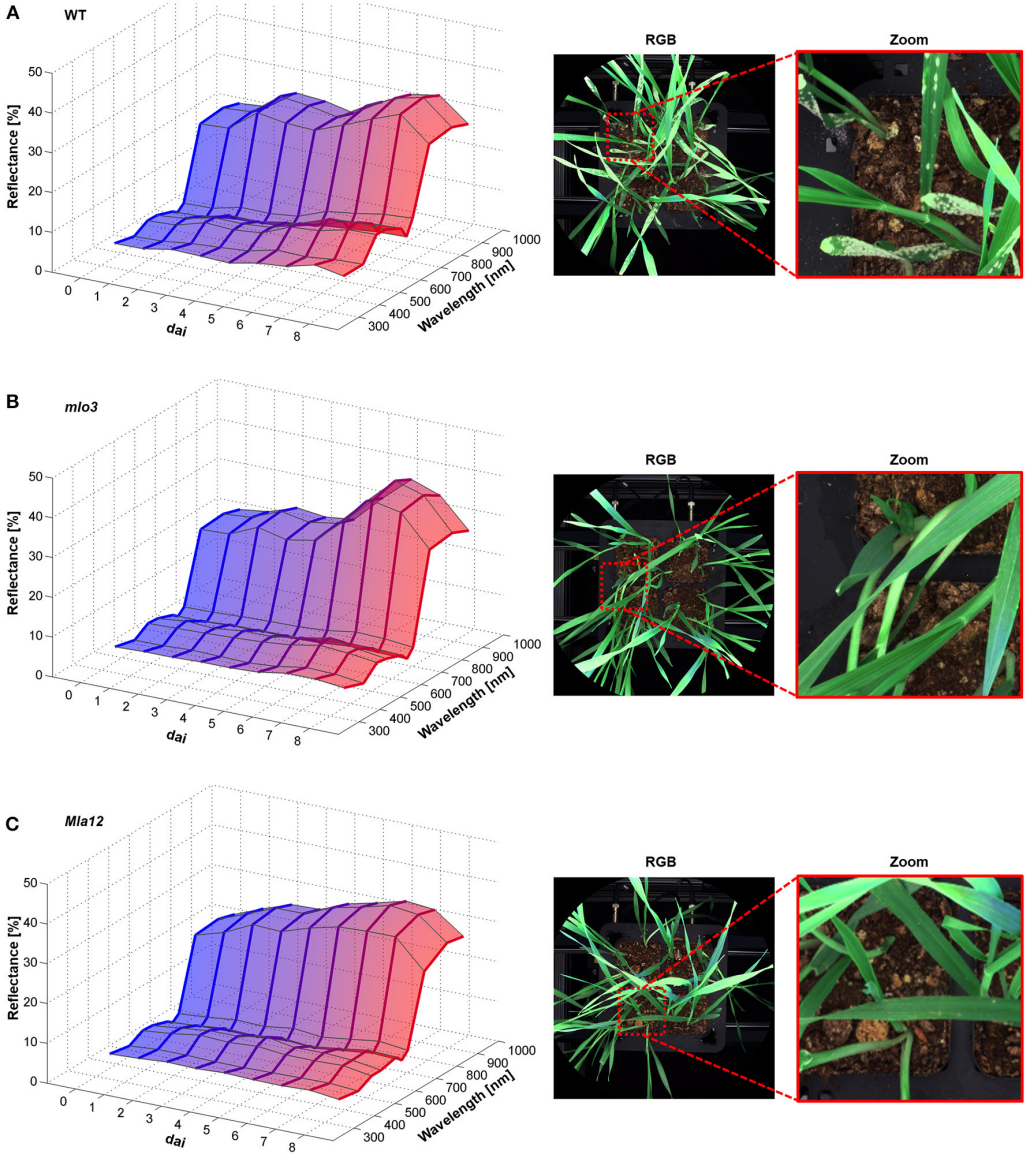
Multispectral signatures of *B. graminis* f.sp. *hordei* inoculated *H. vulgare* leaves cv. Ingrid WT **(A)**, *mlo3* **(B)**, and *Mla12* **(C)** 0–8 dai and corresponding RGB images 7 dai. Susceptible WT leaves showed increased reflectance over the entire spectrum during the experimental period **(A)**. The *mlo3* genotypes showed a slight increase around the green peak and NIR **(B)**. Reflectance intensity of *Mla12* increased especially around 680 nm **(C)** (*n* = 64 × (≥150) biological replicates × technical replicates).

The multispectral reflectance changed over the experimental period specifically for each interaction type (Figure 2). First changes of the reflectance of susceptible cv. Ingrid WT were assessable 2 dai (Figure 2A). The reflectance intensity increased in the VIS range from 380 to 700 nm in accordance with plant growth. Reflectance in the blue range and around 680 nm showed a stronger increase from 4 dai. The NIR range from 700 nm showed a stepwise increase in the reflectance intensity 3 and 5 dai. Powdery mildew pustules were visible from 5 dai and overspread the whole plants 7 dai (Figure 2A). The papilla based resistant *mlo3* genotype showed no relevant changes in the multispectral reflectance until 4 dai (Figure 2B). The reflectance increased in the NIR range from 6 dai. In addition, the intensity increased around 380 and 550 nm. The plants showed no powdery mildew symptoms but several bleached spots. Multispectral signatures of *Bgh* inoculated *Mla12* plants significantly changed from 600 to 680 nm and around 900 nm 4 dai (Figure 2C). A plateau pattern was observed in the spectral range from 550 to 690 nm from 7 dai. The plants show necrotic spots on the leaf surface from 5 to 6 dai.

**Figure 3:**
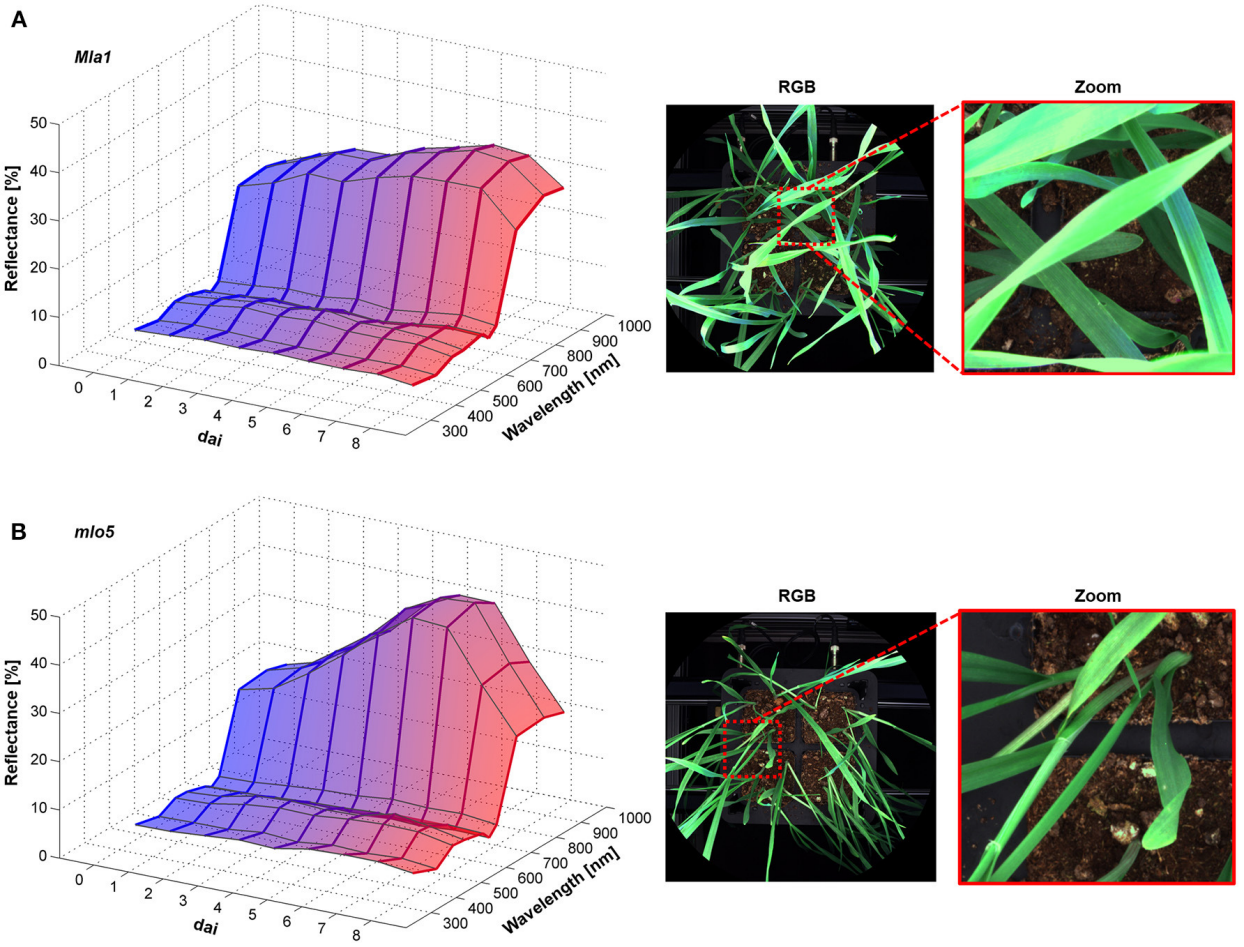
Spectral characteristics of *B. graminis* f.sp. *hordei* inoculated *H. vulgare* leaves cv. Pallas *Mla1* **(A)** and *mlo5* **(B)** 0–8 dai and corresponding RGB images 7 dai (*n* = 64 × (≥150) biological replicates × technical replicates).

The Pallas *Mla1* genotype showed similar changes in the spectral reflectance already 2 dai (Figure 3A). Small necrotic spots were also visible on the leaves from 5 to 6 dai. In contrast, the *mlo5* genotype showed several bleached spots (Figure 3B). The multispectral signature revealed a slight increases in the intensity from 380 to 660 nm. The reflectance intensity in the NIR range showed a continuous increase.

### 3.3. Automatic classification of barley genotypes based on the interaction with *B. graminis* f.sp. *hordei*

**Figure 4:**
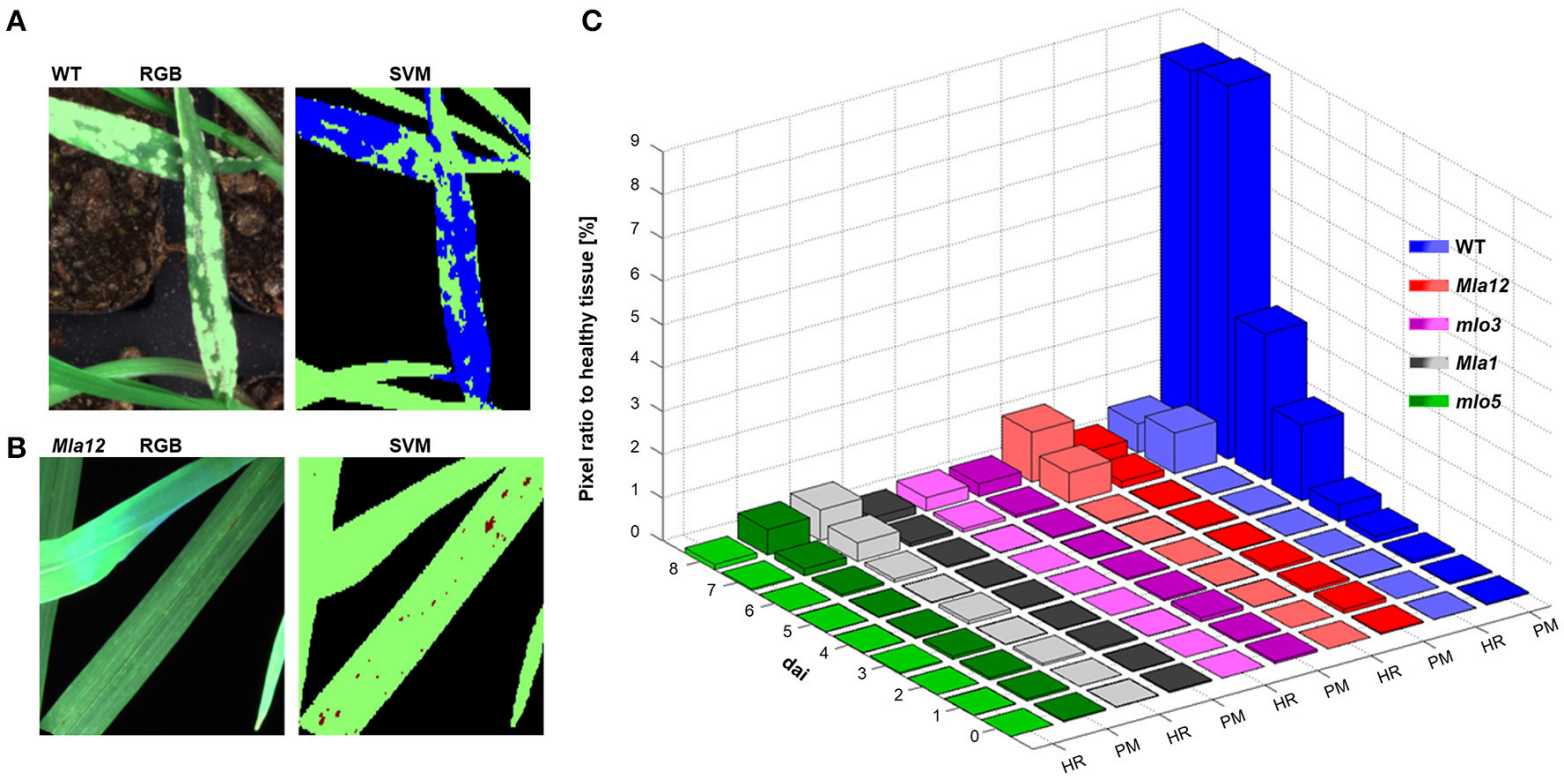
Automatically detected powdery mildew (PM) diseased and HR undergoing pixels applying SVM on multispectral images. In **(A,B)**, representative sections of the multispectral images are illustrated. Healthy tissue is indicated in green pixels and PM diseases tissue in blue pixels **(A)**. Red pixels are indicated tissue undergoing a HR **(B)**. PM and HR pixels are quantified in their ratio to healthy pixels **(C)**. Quantification revealed the susceptible near-isogenic line WT by a high amount of PM diseased pixels from 5 dai. The *Mla* near-isogenic lines can be identified by high amount of HR pixels. Low pixel ratios for both models are shown for *mlo* near-isogenic lines.

Applying the SVM on the multispectral images, the identification of powdery mildew diseased tissue and HR spots was feasible (Figure 4). For the identification of reaction types, two different SVM models were applied. In both analyses, healthy tissue is indicated in green pixels, powdery mildew infested tissue is indicated in blue pixels (Figure 4A), HR spots are indicated in red pixels and non-plant pixels are indicated in black. (Figure 4B).

The ratio determination of powdery mildew diseased pixels and pixels undergoing a HR, revealed a specific pattern which was in accordance with the different barley-powdery mildew interactions (Figure 4C). The quantitative ratios for all investigated near-isogenic lines are shown in Figure 4C. Powdery mildew symptoms can be clearly detected and reach a level of up to 9% of the overall plant pixels. A significant increase in affected pixels was visible at 3 dai with a strong increase till 8 dai. The highest value of the non-susceptibly groups was reached by *mlo5* with below 0.6% at the last day. The determined ratio of HR-reactions reached up to 1.15% for *Mla12* and up to 0.7% for the Pallas *Mla1*. For susceptible Ingrid genotype, up to 0.67% of the pixels were determined as HR reactions.

The detection of the susceptible genotype bases mainly on the powdery mildew-ratio whereas the differentiation between *Mla* and *mlo* resistance is based on the HR-ratio. In the present experiment, a simple threshold based classification was sufficient for an accurate separation of the classes. The confusion matrix, shown in Table 2 summarizes multispectral images of the individual barley-*Bgh* interactions 7 dai. All samples (16 plants per multispectral image) were classified correctly, except of one single *Mla* sample. This sample showed neither powdery mildew symptoms nor a significant number of HR pixels and was therefore assigned to be *mlo* resistant. An overall accuracy of 95% was reached in the present experiment.

**Table 2:** Confusion matrix of automatic prediction of susceptible, *mlo* and *Mla* based resistant barley near-isogenic lines against powdery mildew based on Support Vector Machine analysis of multispectral images 7 dai.

**Predicted**	**Ground Truth**			
	**Susceptible**	***mlo-resistance***	***Mla-resistance***	**Precision**
susceptible	4	0	0	1
*mlo*-resistance	0	8	1	0.89
*Mla*-resistance	0	0	7	1
Recall	1	1	0.88	acc. = 95 %

## 4. Discussion

### 4.1. Temporal dynamics of invertase activity signatures allow early identification of barley-*Bgh* interactions and their functionality is assessable by parallel multispectral imaging

Cw-Inv increased especially in *mlo* and *Mla* resistant near-isogenic lines over the experimental period. An increase in the activity of invertases in *Bgh* inoculated leaves will have several consequences. Beside an increased hydrolysis of sucrose to glucose and fructose, the photosynthesis rate is reduced and several defense genes are activated (Scholes et al., 1994; Both et al., 2005; Swarbrick et al., 2006). Recently, investigations by Brugger et al. (2017) highlighted a decreased photosynthetic rate and an increased non-photochemical quenching of *mlo3* and *Mla1* leaves inoculated with *Bgh*. This is caused by the light energy, reflected as thermal dissipation and not used for photosynthesis. Swarbrick et al. (2006) hypothesized that a reduced photosynthesis rate is induced by increased Cw-Inv activity and play a role in hexoses generation which may supply energy for the defense response and signaling for defense genes against *Bgh*. The increased spectral reflectance intensity 500–700 nm from 48 hai is associated with lower rate in photosynthesis of susceptible WT and resistant *Mla1* and *Mla12*. A specific increase of Cw-Inv activity during plant-pathogen interaction was also observed in different systems and trophic styles (Proels and Hückelhoven, 2014). This supports Cw-Inv as a promising biomarker, but several signals are converged at the site of Cw-Inv. Therefore, this potential biomarker must be evaluated for every specific plant-pathogen system. In this study, Cw-Inv and Cyt-Inv showed to be significantly different between the investigated plant-pathogen systems at least during two experimental time points. The spectral reflectance showed similar patterns for the specific interaction and allowed an accurate characterization of the interaction type. Differences on the “omic level” can be induced by their different signal pathways e.g., *Mla1* induces *Bgh* race-specific resistance via a RAR1 independent pathway (Schulze-Lefert and Vogel, 2000; Bieri et al., 2004).

Specific activities of the tested invertase isoenzymes was significant lower in *Bgh* inoculated WT compared to the corresponding non-inoculated leaves already 0.5 hai. Furthermore, the continuous increase of Vac-Inv activity from 48 hai was in accordance with increased reflectance around 365, 460, and 670 nm of susceptible WT, which is characteristic for powdery mildew pathogenesis (Kuska et al., 2015, 2017; Wahabzada et al., 2015). Such early phenomena was investigated by Nielsen et al. (2000). They have shown an extracellular proteinaceous matrix from the conidia body by electron microscopy already 1 hai. They proposed even an earlier interaction between plant and conidia, because they identified that conidia can uptake low-molecular-weight compounds before germination. This makes *Bgh* conidia capable of signal- recognition and respond to the host, immediately after the first contact (Nielsen et al., 2000). In this context, it was also shown that cell wall carbohydrates contribute to penetration resistance (Ellinger et al., 2013). Later increased invertase activities in this study are similar to results of Scholes et al. (1994); Both et al. (2005); Swarbrick et al. (2006) and are important to facilitate nutrition uptake by the *Bgh* haustoria which prefer glucose (Whipps and Lewis, 1981). In addition, the increased spectral reflectance in the green and red range are in accordance with studies by Scholes et al. (1994). They hypothesized a reduced photosynthesis activity due to increased invertase activity, because the increased carbohydrate concentration down regulates the Calvin cycle. These would affect plant development and architecture, which are indicated in the NIR range (Gates et al., 1965; Slaton et al., 2001). But, increasing reflectance between 750 and 1,000 nm in this study was mainly caused by plant growth and leaf overlapping. The decreased reflectance intensity 8 dai is caused due to hang down and overlapping of the barley leaves. Thus, the leaves were in a different height and angle composed to earlier experimental days. This can be avoided using a plant fixation which keep the leaves in position at every day time and will reduce the effect of increasing reflectance intensity over the whole spectrum and experimental period (Mahlein et al., 2012). Further solution, which consider the leaf angle could be the implementation of 3D models for the normalization of spectral reflectance (Behmann et al., 2016). Different state-of-the-art optical approaches and machine learning applications for the estimation of disease severity were realized on the leaf level (Bock et al., 2010; Rumpf et al., 2010; Pethybridge and Nelson, 2015; Kuska et al., 2017; Thomas et al., 2017). Thomas et al. (2018a) established a mini-plot facility in the greenhouse for high-throughput identification and quantification of powdery mildew tolerant barley lines using hyperspectral imaging in the VIS range. For detailed review of limitations and solution statements for spectral imaging in plant breeding processes, we refer to Kuska and Mahlein (2018) and Thomas et al. (2017). In our study, the quantification of diseased and HR pixels was feasible on whole plants using SVM on multispectral images. This enables the characterization of barley-powdery mildew interaction types with a high precision and shows the potential of machine learning methods for high-throughput resistance screening (Behmann et al., 2015). In further trials, the trained model can be applied to identify and characterize unknown genotypes as *mlo* or *Mla* resistant, even if the causing locus is unknown so far. For further models, the specific crops and pathogenesis must be investigated. Therefore, the use of specific fungal isolates is essential to identify race-specific resistances e.g., against the wheat stem rust isolate Ug99. Here the isolate overcame wheat resistances and common biological markers are now limited or inoperative (Singh et al., 2011). The here presented technique and method has high potential to identify new promising parental candidates for present and future breeding purposes in a fully automated manner. However, a direct differentiation of the *mlo* and *Mla* loci was not realized by the multispectral imaging, which shows the current limitation for practical breeding processes. For such a detailed phenotyping, the coherency of hyperspectral reflectance signatures with physiological and “omic data” must be systematically investigated (Arens et al., 2016; Leucker et al., 2017; Kuska and Mahlein, 2018). New developed markers and resulted new breeding lines can be then tested under different environmental conditions and will be analyzed and assessed by multispectral imaging.

In summary, this study represents a successful proof-of-concept for effective and efficient screening of barley-powdery mildew interaction types in a controlled environment with high-throughput solutions. Data analysis can be highly improved by machine learning approaches. In addition to reduced labor intensity, a pixelwise disease and HR spot estimation was enabled by a SVM which allows a precise barley-powdery mildew interaction type prediction. Consequently, multispectral imaging can be used for high-throughput plant resistance screenings to identify resistant plants and to differentiate them in a controlled environment. The distinct temporal dynamics of changes in activity signatures of invertase isoenzymes can be used for early identification of barley-*Bgh* interactions, which are assessed on functionality by parallel multispectral imaging. In future approaches, multispectral imaging will be established for different environmental scenarios to analyze the stability of plant resistance in combination with abiotic stress factors. In these scenarios, the combination of metabolic and phenomic profiles will be highly informative.

## Author contributions

MK, JB, DG, TR, and A-KM designed the study. MK, JB, and A-KM drafted the manuscript. MK and DG carried out multispectral measurements. MK manually analyzed multispectral images and determined and assessed enzyme kinetics. JB adapted and applied the SVM on multispectral images to identify and quantify plant-pathogen interactions and Welchs *t*-test on enzyme activities. MK, JB, DG, TR, and A-KM interpreted the data. All authors read and approved the final manuscript.

### Conflict of interest statement

The authors declare that the research was conducted in the absence of any commercial or financial relationships that could be construed as a potential conflict of interest.
